# Optimism and Mortality in Older Men and Women: The Rancho Bernardo Study

**DOI:** 10.1155/2016/5185104

**Published:** 2016-03-03

**Authors:** Ericha G. Anthony, Donna Kritz-Silverstein, Elizabeth Barrett-Connor

**Affiliations:** ^1^Department of Family Medicine and Public Health, University of California San Diego, La Jolla, CA 92093-0628, USA; ^2^Graduate School of Public Health, San Diego State University, San Diego, CA 92182, USA

## Abstract

*Purpose.* To examine the associations of optimism and pessimism with all-cause, cardiovascular disease (CVD), coronary heart disease (CHD), and cancer mortality in a population-based sample of older men and women followed ≤12 years.* Methods.* 367 men and 509 women aged ≥50 from the Rancho Bernardo Study attended a 1999–2002 research clinic visit when demographic, behavioral, and medical history were obtained and completed a 1999 mailed survey including the Life Orientation Test-Revised (LOT-R). Mortality outcomes were followed through 2012.* Results.* Average age at baseline was 74.1 years; during follow-up (mean = 8.1 years), 198 participants died, 62 from CVD, 22 from CHD, and 49 from cancer. Total LOT-R, optimism and pessimism scores were calculated. Participants with the highest optimism were younger and reported less alcohol use and smoking and more exercise. Cox proportional hazard models showed that higher total LOT-R and optimism, but not pessimism scores, were associated with reduced odds of CHD mortality after adjusting for age, sex, alcohol, smoking, obesity, physical exercise, and medication (HR = 0.86, 95% CI = 0.75, 0.99; HR = 0.77, 95% CI = 0.61, 0.99, resp.). No associations were found for all-cause, CVD, or cancer mortality.* Conclusions.* Optimism was associated with reduced CHD mortality in older men and women. The association of positive attitudes with mortality merits further study.

## 1. Introduction

Numerous studies report significant associations between optimism or pessimism and various health outcomes including cardiovascular disease (CVD), coronary heart disease (CHD), myocardial infarction, and cancer; most report that optimism is protective whereas pessimism is associated with increased risk of disease [1–6]. The few clinical studies that examined the association between optimism or pessimism and mortality report less consistent results. For example, a study of 238 US male and female cancer patients aged 30 years and older suggested that pessimism was a mortality risk factor only for younger patients [7], while a predominantly male French cohort of 101 cancer patients, aged 35 to 81 years, reported greater risk of death for pessimistic as compared to optimistic patients one year after diagnosis [6]. A recent study by Chang et al. showed statistically significant sex differences in the reporting of psychological outcomes: men were more inclined to report positive psychological outcomes for* self rather than for others* and also more likely to report negative psychological outcomes for* others rather than for self* [8].

Only two population-based studies have reported the association of optimism or pessimism with mortality. Among 97,253 women aged 50 to 79 years from Women's Health Initiative who were followed up by mail for eight years, optimism was associated with a reduction of 14% for total, 24% for CVD, and 30% for CHD-related mortality after adjusting for traditional and lifestyle risk factors such as age, hypertension, BMI, smoking, alcohol use, and physical activity [4]. The Arnhem Elderly Study of 999 Dutch men and women aged 65 to 85 years followed up for nine years reported protective effects of optimism on all-cause and CVD mortality in men after adjusting for risk factors, including lifestyle and medical history, whereas, after adjusting for the same risk factors in women, the protective effect of optimism was found only for CVD mortality [9]. Analysis of a 15-year follow-up of men only from the Zutphen Study showed that optimism was associated with lower risk of cardiovascular death [10] as well as healthier lifestyle and dietary habits, suggesting that lower levels of optimism may influence behavioral choices leading to cardiovascular death [11]. However, there has been no US population-based study of both older men and women who were followed up for 10–12 years.

The purpose of this report was to examine the association of optimism and pessimism with all-cause and cause-specific mortality in the Rancho Bernardo Study, a large population-based sample of community-dwelling older men and women. Given the associations of optimism and pessimism with multiple diseases and the inconsistency in the literature relating to mortality, it is important to determine how optimism and pessimism are associated with mortality and whether there are sex differences in these associations.

## 2. Materials and Methods

### 2.1. Participants

**Figure 1:**
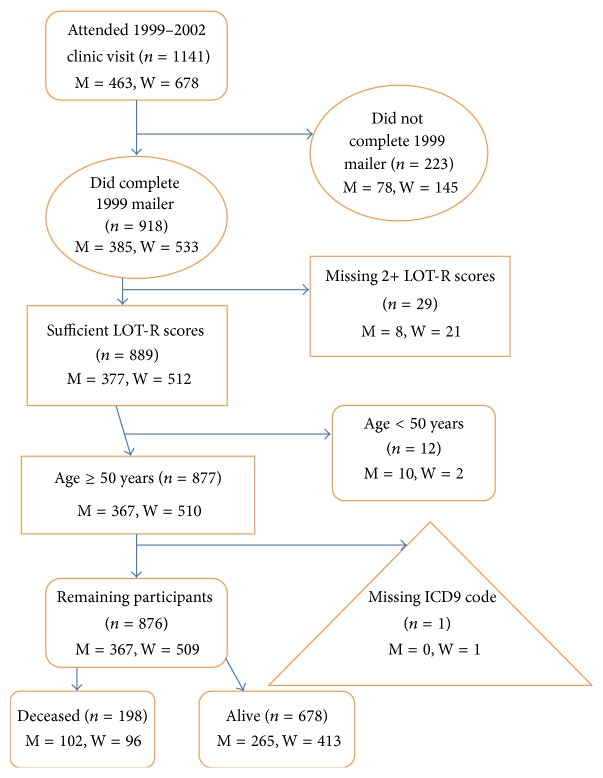
Study participant flow chart.

Between 1972 and 1974, the Rancho Bernardo Heart and Chronic Disease Study enrolled 82% (*n* = 6629) of residents aged 30 to 79 years from the Southern California community of Rancho Bernardo. These participants have been followed up with periodic clinic visits and yearly mailed surveys; death certificates were obtained for all decedents. Between 1999 and 2002, 463 men and 678 women (*n* = 1141) participated in a follow-up research clinic visit. In 1999, a mailed survey including the Life Orientation Test-Revised (LOT-R) questionnaire used to assess optimism and pessimism was mailed to all participants. Participants for this study were members of the Rancho Bernardo cohort who attended the 1999–2002 clinic visits, responded to the 1999 mailed LOT-R questionnaire, and were followed up through 2012. After excluding the 78 men and 145 women (*n* = 223) who did not complete the LOT-R questionnaire, 8 men and 21 women (*n* = 29) missing two or more LOT-R responses, 10 men and 2 women (*n* = 12) younger than age 50 at the time of this clinic visit, and 1 woman missing a death certificate, there remained a total of 367 men and 509 women (*n* = 876) who formed the cohort for this report. Participants were followed up through 2012, the last year for which complete mortality data was available; at that time, 102 men and 96 women (*n* = 198) were deceased; 265 men and 413 women (*n* = 678) were alive (Figure 1).

This study was approved by the Human Research Protections Program at the University of California, San Diego. All participants gave written informed consent prior to participation.

### 2.2. Procedures

At the 1999–2002 clinic visit, height and weight were measured in participants wearing light clothing without shoes and used for calculation of body mass index (BMI, kg/m^2^) as an estimate of obesity. Waist circumference was measured at the bending point and hip girth was measured at the widest point for calculation of waist-hip ratio (WHR) as an estimate of central adiposity. Two blood pressure measures were obtained 5 minutes apart by a nurse trained in the Hypertension Detection and Follow-up Program (HDFP) protocol after participants had been seated quietly for five minutes, using the average of the two systolic and diastolic measures [12].

A trained interviewer used a standardized interview to obtain information on current marital status (no/yes), cigarette smoking history (never/past/current), and exercise 3 or more times per week (no/yes). Alcohol use during an average week (grams/week) was calculated based on the number of beers and glasses of wine and drinks of hard liquors and liqueurs per week. Participants were asked about their medical history including physician's diagnosis of hypertension, diabetes, heart attack, transient ischemic attack (TIA), stroke, angina, and cancer. Participants were also queried about current medication use including antihypertensives and angina treatment and cholesterol-lowering and diabetes medications. Women were also asked about hormone replacement therapy (HRT) use and duration. Current medications were validated by a nurse who examined pills and containers brought to the clinic for that purpose. The Medical Outcomes Short-Form Health Survey (SF-12) is a 12-item, self-report measure of functional health and well-being from the participant's point of view [13]. This scale has been reported to have test-retest (2-week) correlations of 0.89 and 0.76, respectively, for the 12-item Physical Component Summary and the 12-item Mental Component Summary [13].

In 1999, a mailed survey included the Life Orientation Test-Revised (LOT-R), a widely used, 10-item, validated questionnaire assessing dispositional optimism that consists of 3 items assessing optimism, 3 items assessing pessimism, and 4 filler items [14]. The total LOT-R score takes into account the relative contributions of optimism and pessimism whereas all of the subscales only assess a single dimension. Responses are given on a 0–4 scale ranging from Strongly Agree to Strongly Disagree [14]. An example of an optimism question is, “In uncertain times, I usually expect the best” and an example of a pessimism question is, “If something can go wrong for me, it will” [14]. This scale has been reported to have internal reliability of 0.78 and test-retest reliability of 0.79 at 28 months [14]. Internal consistency for total LOT-R in this sample was 0.73 based on Cronbach's *α* coefficient. In addition to the total LOT-R score, the authors also separately examined the 3 optimism items (optimism subscale) and the 3 pessimism items (pessimism subscale) to determine whether there were differences across these subscales and the total score.

Death certificates were obtained for all decedents and cause of death was coded by a certified nosologist using the International Classification of Disease, Ninth Revision (ICD-9). Cancer deaths included codes 140–239, CVD deaths included codes 401–414, 426–438, and 440–448, and CHD deaths included codes 410–414.

### 2.3. Statistical Analysis

Total LOT-R score was calculated by reverse scoring the three optimism items and adding this value to the sum of the three pessimism items to obtain a score ranging from 0 to 24; higher total LOT-R scores indicate greater optimism [15]. Filler items were not used when calculating the total score. A separate subscale score for optimism was calculated by summing the three reverse scored optimism items and a separate subscale score for pessimism was calculated by summing the scores for the three pessimism items. For both optimism and pessimism subscales, if one item was unanswered, it was given the mean value of the two answered optimism or pessimism items; higher scores indicated greater optimism or pessimism. The total LOT-R score measures the balance of optimism versus pessimism whereas the optimism subscale measures optimism only and the pessimism subscale measures pessimism only. Based on data distributions, total LOT-R scores were divided into quartiles and the subscales were each divided into tertiles. Data were analyzed for both sexes combined and also stratified by sex. Descriptive statistics were calculated and reported as rates for categorical data and means (± standard deviations) for continuous data. Comparisons were performed for categorical variables using chi-square tests and for continuous variables using independent *t*-tests. LOT-R scores were divided into quartiles of increasing optimism based on the total sample (0–15, 16-17, 18-19, and 20–24); comparisons between quartiles were made for age, behaviors such as exercise, alcohol use, smoking, and other potential confounders using age-adjusted univariate logistic regression for categorical variables and age-adjusted univariate linear regression for continuous variables. Because all analyses yielded similar results for men and women, only the results using data from both sexes combined are shown. Variables for which differences between quartiles were obtained where *p* < 0.20 were included as covariates in later multivariable analyses. Forward stepwise Cox proportional hazard models were used to assess the association between continuous total LOT-R score and each of four main mortality outcomes: all-cause, cancer, CVD, and CHD mortality. For variables that did not meet the proportional hazards assumption (*p* value ≤ 0.05), the time/variable interaction term was also included in the final model. Time was measured from date of 1999–2002 clinic visit to date of last contact or date of death. Model 1 examined the unadjusted associations of total LOT-R score with each mortality outcome. Model 2 included total LOT-R and age. Model 3 included Model 2 variables with sex added as a covariate. Model 4 included Model 3 variables with average alcohol use per week, smoking status, WHR, and exercise added. Model 5 included Model 4 variables plus angina, cholesterol-lowering, and diabetic medications. Hazard ratios, 95% confidence intervals, and *p* values are reported. All analyses were conducted using SAS version 9.2 (SAS Institute, NC); *p* value ≤ 0.05 was considered statistically significant. The sample size of 876 participants had greater than 80% statistical power for detecting an association with 95% confidence [16].

## 3. Results

Table 1 shows descriptive statistics for men and women combined and separately for each sex. Average age was 74.1 ± 9.7 years (men 74.3 ± 9.3 years and women 74.1 ± 10.1 years). Compared to women, men had significantly higher average alcohol consumption per week (50.6 g versus 79.6 g, *p* < 0.001, resp.) and higher rates of medication use for angina (*p* = 0.001), cholesterol (*p* < 0.001), and diabetes (*p* = 0.02). Participants were followed up for up to 12 years for an average of 8.1 years. There were no significant differences in total LOT-R score or optimism and pessimism subscale scores between men and women (*p* = 0.99). During follow-up there were 198 deaths due to all causes, 49 due to cancer, 62 due to CVD, and 22 due to CHD. All-cause mortality and CHD mortality were significantly higher in men than in women (27.8% versus 18.9%, *p* < 0.001, and 4.4% versus 1.2%, *p* = 0.04). There were no significant sex differences in cancer (6.5% versus 4.9%, *p* = 0.85) or CVD mortality (8.4% versus 6.1%, *p* = 0.76). Other comparisons for men and women are shown in Table 1.

Age and age-adjusted comparisons of covariates by quartile of LOT-R scores for men and women combined are presented in Table 2. There were significant differences in age by quartile of total LOT-R; those with the highest quartile had the lowest mean age while those with the lowest quartile had the highest mean age (*p* ≤ 0.001). There were significant differences by quartile of total LOT-R in rates of smoking (*p* = 0.05), use of cholesterol-lowering medications (*p* = 0.02), and average alcohol use per week (*p* = 0.02).

**Figure 2:**
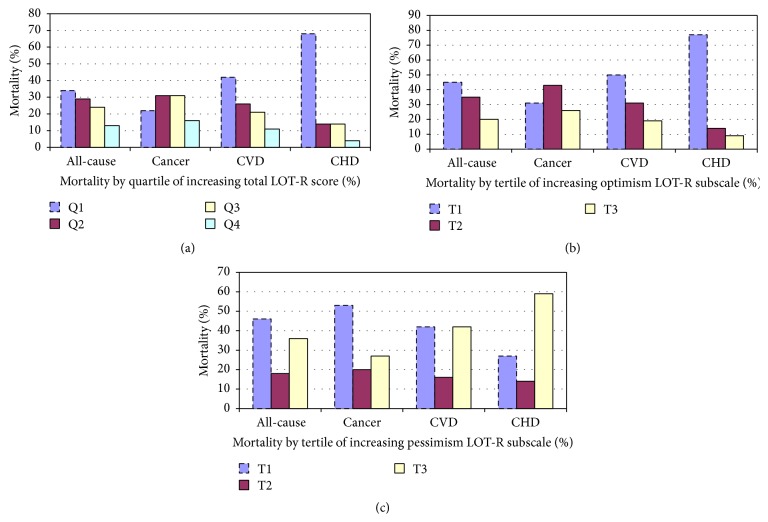
(a) Unadjusted comparisons of overall and cause-specific mortality^+^ by quartile of total LOT-R; Rancho Bernardo, CA, 1999–2012 (*n* = 876).* Reference*: all-cause mortality, *p* = 0.01 when Q1 compared to Q4; cancer mortality, *p* = 0.05 when Q1 compared to Q4; CHD mortality, *p* = 0.001 when Q1 compared to Q4; ^+^all-cause, cancer, CVD, and CHD mortality are present among those who died. (b) Unadjusted comparisons of overall and cause-specific mortality^+^ by tertile of optimism subscale score; Rancho Bernardo, CA, 1999–2012 (*n* = 876).* Reference*: cancer mortality, *p* = 0.03 when T1 compared to T3; CHD mortality, *p* = 0.003 when T1 compared to T3. (c) Unadjusted comparisons of overall and cause-specific mortality^+^ by tertile of pessimism subscale score; Rancho Bernardo, CA, 1999–2012 (*n* = 876).* Reference*: all-cause mortality, *p* = 0.002 when T1 compared to T3.

Figures 2(a)–2(c) show unadjusted comparisons of all-cause, cancer, CVD, and CHD mortality percentages by quartile of total LOT-R and by tertiles of optimism and pessimism subscales scores. All-cause, CVD, and CHD mortality decreased with increasing quartile of total LOT-R (Figure 2(a)) and increasing tertile of optimism subscale score (Figure 2(b)). There were significant differences for all-cause, cancer, and CHD mortality between the lowest and highest quartiles of total LOT-R and significant differences between the lowest and highest tertiles of the optimism subscale scores for cancer and CHD deaths. Conversely, CHD mortality increased with increasing tertile of pessimism subscale (Figure 2(c)), with significant differences between the lowest and highest tertile of pessimism subscale for all-cause mortality.

Table 3 shows the associations of total LOT-R and optimism and pessimism subscale scores with mortality, after adjusting for covariates. Prior to adjusting for covariates, there were significant associations between total LOT-R score and lower rates of all-cause (*p* = 0.002), CVD (*p* = 0.004), and CHD mortality (*p* = 0.003). After adjusting for age, odds of CHD mortality were 15% lower for those with higher optimism (HR = 0.85, 95% CI = 0.74, 0.97), a difference that remained significant after further adjustment for sex, alcohol use, smoking status, waist-hip ratio (WHR), exercise status, and use of angina treatment and cholesterol-lowering and/or diabetic medications (HR = 0.86, 95% CI = 0.75, 0.99). Associations of total LOT-R with all-cause and CVD mortality became nonsignificant after adjustment for age. There were no significant associations between total LOT-R and cancer mortality either before or after adjustment for covariates. Similar patterns were found for the optimism subscale score, which was significantly associated with decreased risk of CHD before and after adjustment for age, sex, alcohol use, smoking status, WHR, exercise status, and use of angina, cholesterol-lowering, and/or diabetic medications (HR = 0.77, 95% CI = 0.61, 0.99). The optimism subscale score was not significantly associated with all-cause, cancer, or CVD mortality. The pessimism subscale score also was not associated with odds of all-cause or cause-specific mortality after adjusting for age and/or other covariates.

## 4. Discussion

In both sexes, higher optimism, whether based on total LOT-R score or based on optimism subscale score, was associated with 14% and 23% lower risk, respectively, of CHD mortality. Although participants with higher optimism had healthier lifestyle behaviors, these associations were independent of age, sex, lifestyle variables (alcohol use, smoking status, obesity, and exercise), and medication use; adjustment for these variables did not alter the results. After adjustment for covariates there was no association of optimism with CVD, all-cause, or cancer mortality and no association of pessimism with all-cause or cause-specific mortality. Results of this study are important, as, to our knowledge, this is the largest population-based US study of optimism and mortality that includes both older men and women.

These results are in accord with those from Women's Health Initiative, which reported a significant association between higher levels of optimism based on total LOT-R and a 30% reduction in CHD mortality for white women after adjusting for age and other potential confounders [4]. However, our study included both sexes and found similar reductions of ~14% for CHD mortality for analyses including both men and women and in other analyses stratified by sex.

In contrast to results from Women's Health Initiative, our study did not find an association between higher levels of optimism and reduced odds of all-cause and CVD mortality [4], nor was pessimism associated with increased odds of cancer mortality as shown in some prior clinical studies [6, 7]. However, participants in our study were older, had higher socioeconomic status, and had health insurance compared to previous studies. Associations of pessimism with poor outcomes disappeared after adjusting for age and other covariates, suggesting a relatively large role of age and lifestyle factors on all-cause or CVD deaths. Our results also differed from both the Arnhem Elderly Study where optimism was protective for all-cause and CVD mortality and the Zutphen Study where optimism was protective for CVD mortality, but this is most likely due in part to the fact that scales other than the LOT-R were used to assess dispositional optimism [9–11].

Results of this study have clinical utility as they suggest that a personality characteristic, namely, optimism, is protective against CHD mortality. The biological and psychological mechanism of action between optimism, pessimism, and mortality are complex and not fully understood. Our participants with higher optimism scores had healthier behaviors; those in the highest optimism quartile were younger, drank less per week, smoked less, and exercised more, and a greater proportion were married compared to participants in the three lower optimism quartiles. Thus, optimism may exert indirect effects on health via a healthier lifestyle or marital status. However, optimism also had an effect on health independent of lifestyle and other covariates. Participants in the highest optimism quartile also had lower systolic blood pressure and lower rates of hypertension, diabetes, and heart disease, and fewer reported taking medications for hypertension, angina, and cholesterol. Others have shown that optimism is associated with lower levels of inflammation [17] and stress and negative emotions [18, 19]. Thus, it is plausible that optimism exerts a beneficial effect by improved physiologic parameters such as reduced inflammation [17] or biological side effects of stress (such as hypertension, diabetes, and heart disease), and negative emotions, thereby contributing to reduced mortality in more optimistic persons [18, 19]. While recent studies have shown associations between increased levels of inflammatory biomarkers and increased risk of CHD [20, 21], it is not clear if this is unique to CHD or applies to CVD in general [21].

Several limitations and strengths of this study must be considered. Given the older age of our study participants, it is conceivable that an association of optimism or pessimism with mortality was missed because participants had died before the follow-up assessment. Our study included participants who completed both the clinic visit and the mailed survey. Comparisons showed that those who did not complete the mailed survey had a higher proportion of men (65%), fewer married individuals (63.7% versus 70.2%), and lower rates of exercise (64.1% versus 72.1%) and drank on average less alcohol per week (53.9 g versus 67.2 g). Thus, survival bias cannot be excluded. Self-reported responses on the LOT-R introduce the possibility of misreporting. Due to the small number of cases in each analyzed mortality subgroup, we cannot exclude the possibility that low power may have reduced the ability to detect differences in mortality, although the association of optimism with reduced CHD mortality argues against this. Participants from the Rancho Bernardo Study are white and middle-class with relatively good access to healthcare. Thus, these results may not be generalizable to different ethnicities, lower socioeconomic status, or limited healthcare. On the other hand, this homogeneity means that the associations of optimism and pessimism with mortality may be less confounded by these differences. A major strength of this study includes its prospective design with follow-up for mortality over 10–12 years.

## 5. Conclusion

In conclusion, participants with higher optimism had healthier behaviors, but optimism was associated with reduced risk of CHD mortality independent of age, sex, and behaviors including alcohol use, smoking status, obesity, exercise, and medication use. These associations suggest similar direct and indirect effects of optimism on CHD mortality in men and women. Additional studies including both men and women with longer follow-up are necessary to further explore both sex and cause-specific nature of these associations.

## Figures and Tables

**Table 1:** Unadjusted comparison of LOT-R scores and behaviors and other covariates for men and women, Rancho Bernardo, CA, 1999–2002 (*N* = 876).

	All (*n* = 876)	Men (*n* = 367)	Women (*n* = 509)	*p* value^*∗*^
	Mean (SD)	Mean (SD)	Mean (SD)	
Age (yr)	74.1 (9.7)	74.3 (9.3)	74.1 (10.1)	0.78
Follow-up (yr)	8.1 (2.7)	7.8 (2.9)	8.3 (2.5)	0.01
Alcohol use (avg g/wk)	62.7 (78.9)	79.6 (92.0)	50.6 (65.4)	**<0.001**
Duration of smoking (yr)	23.7 (15.9)	23.7 (15.5)	23.8 (16.2)	0.96
WHR	0.87 (0.09)	0.95 (0.05)	0.81 (0.06)	**<0.001**
BMI (kg/m^2^)	26.3 (4.2)	27.0 (3.7)	25.7 (4.5)	**<0.001**
SBP (mmHg)	139.7 (20.3)	138.9 (20.3)	140.3 (20.3)	0.33
DBP (mmHg)	78.2 (9.5)	78.8 (10.1)	77.7 (9.0)	0.09
Duration HRT (yr)	—	—	14.8 (12.3)	—
LOT-R total	17.1 (3.2)	17.1 (3.3)	17.1 (3.2)	0.99
Optimism subscale	8.5 (1.7)	8.5 (1.7)	8.6 (1.7)	0.69
Pessimism subscale	3.5 (1.9)	3.4 (1.9)	3.5 (1.9)	0.71

	*N* (%)	*N* (%)	*N* (%)	

Mortality, all-cause	198 (22.6)	102 (27.8)	96 (18.9)	**<0.001**
Cancer	49 (5.6)	24 (6.5)	25 (4.9)	0.85
CVD	62 (7.1)	31 (8.4)	31 (6.1)	0.76
CHD	22 (2.5)	16 (4.4)	6 (1.2)	**0.04**
HRT (ever)	—	—	264 (51.9)	—
Smoking status				**0.003**
Never	394 (45.0)	141 (38.4)	253 (49.7)	
Past	446 (50.9)	211 (57.5)	235 (46.2)	
Current	36 (4.1)	15 (4.1)	21 (4.1)	
Exercise ≥ 3x/wk (% yes)	632 (72.1)	274 (74.7)	358 (70.3)	0.16
Marital status (% yes)	615 (70.2)	310 (84.5)	305 (59.9)	**<0.001**
BP low meds (% yes)	302 (34.5)	134 (36.5)	168 (33.0)	0.27
Angina meds (% yes)	25 (2.9)	19 (5.2)	6 (1.2)	**0.001**
Chol low meds (% yes)	230 (26.3)	119 (32.4)	111 (21.8)	**<0.001**
Diabetes meds (% yes)	40 (4.6)	24 (6.5)	16 (3.1)	**0.02**
Hypertension (% yes)	561 (64.0)	244 (66.5)	317 (62.3)	0.18
Diabetes (% yes)	55 (6.3)	27 (7.4)	28 (5.5)	0.22
Heart attack (% yes)	33 (3.7)	17 (4.6)	16 (3.1)	0.17
TIA (% yes)	58 (6.6)	35 (9.5)	23 (4.5)	**0.002**
Stroke (% yes)	10 (1.1)	9 (2.5)	1 (0.02)	**0.001**
Angina (% yes)	50 (5.7)	38 (10.4)	12 (2.4)	**<0.001**
Cancer (% yes)	189 (21.6)	84 (22.9)	105 (20.6)	0.26

^*∗*^Reference: sex differences: *t*-test is used for continuous variables; *χ* ^2^ is used for categorical variables; bold indicates *p* value ≤ 0.05.

**Table 2:** Age and age-adjusted covariates comparison by LOT-R score quartiles in both sexes, Rancho Bernardo, CA, 1999–2002 (*N* = 876).

	Q1 (0–15)	Q2 (16-17)	Q3 (18-19)	Q4 (20–24)	
	(*n* = 242)	(*n* = 234)	(*n* = 230)	(*n* = 170)	*p* value
	Mean	Mean	Mean	Mean	
Age (yr)	76.6	74.4	73.8	70.9	**<0.001** ^**+**^
Follow-up (yr)	7.7	7.8	8.3	8.7	0.06
Alcohol use (avg g/wk)	64.4	56.9^**∗∗∗**^	75.6^**∗∗∗**^	50.8	**0.02**
Duration of smoking (yr)	24.8	24.3	24.6	19.4	0.15
WHR	0.86	0.87	0.87	0.87	0.75
BMI (kg/m^2^)	26.0	26.7	26.0	26.3	0.26
SBP (mmHg)	140.5	141.7	139.3	136.6	0.41
DBP (mmHg)	77.3	78.8	77.9	78.8	0.59
SF-12					
PCS	43.2^∧∧**∗**^	44.7^**∗**^	47.5	50.8	**<0.001**
MCS	51.7^∧**∗**^	53.0^**∗**∧∧^	55.7	57.7	**<0.001**

	%	%	%	%	

Sex (male)	44.2	35.5^**∗∗∗**^	43.9	44.7	0.14
Smoking status (ever)	55.4	61.1^**∗∗∗**^	55.2	45.9	**0.05**
Exercise ≥ 3x/wk (% yes)	68.6	68.8	75.2	77.6	0.14
Marital status (% yes)	69.8	68.4	70.9	72.4	0.62
BP low meds (% yes)	38.8	33.9	33.3	28.2	0.82
Angina meds (% yes)	6.6	2.6	0.01	0	0.06
Chol low meds (% yes)	23.1	30.3	30.4^**∗∗∗**^	19.4	**0.02**
Diabetes meds (% yes)	2.5	7.7^**∗∗**^	4.3	3.5	0.06
Hypertension (% yes)	68.6	65.4	61.7	58.8	0.93
Diabetes (% yes)	5.0	9.0	6.1	4.7	0.29
Heart attack (% yes)	5.4	3.0	3.5	2.9	0.75
TIA (% yes)	5.8	6.8	6.5	7.6	0.77
Stroke (% yes)	0.01	0.02	0.01	1.2	0.83
Angina (% yes)	6.2	4.3	7.4	4.7	0.43
Cancer (% yes)	19.4	22.6	23.0	21.2	0.88

Reference: compared to Q3 only ^∧^ *p* < 0.001, ^∧∧^ *p* < 0.01, and compared to Q4 only ^**∗**^ *p* < 0.001, ^*∗∗*^ *p* < 0.01, and ^*∗∗∗*^ *p* < 0.05; ^+^unadjusted age; total LOT-R scored 0–24 by increasing optimism by quartile; bold indicates *p* value < 0.05.

**Table 3:** Associations of continuous LOT-R score with mortality in both sexes, Cox proportional hazard modeling, Rancho Bernardo, CA, 1999–2002 (*N* = 876).

	All-cause mortality		Cancer mortality		CVD mortality		CHD mortality	
	HR (95% CI)	*p* value	HR (95% CI)	*p* value	HR (95% CI)	*p* value	HR (95% CI)	*p* value
Total LOT-R score								
Model 1	**0.94 (0.90, 0.98)**	**0.002**	1.00 (0.92, 1.09)	0.99	**0.90 (0.84, 0.97)**	**0.004**	**0.85 (0.77, 0.95)**	**0.003**
Model 2	0.98 (0.93, 1.02)	0.32	1.03 (0.94, 1.13)	0.53	0.94 (0.87, 1.02)	0.14	**0.85 (0.74, 0.97)**	**0.02 **
Model 3^*∗*^	0.98 (0.94, 1.02)	0.36	1.03 (0.94, 1.13)	0.53	0.94 (0.87, 1.02)	0.16	**0.86 (0.76, 2.98)**	**0.02 **
Model 4	0.99 (0.94, 1.03)	0.50	1.03 (0.94, 1.13)	0.48	0.95 (0.88, 1.03)	0.22	**0.87 (0.76, 0.99)**	**0.04**
Model 5	0.99 (0.94, 1.03)	0.53	1.04 (0.94, 1.14)	0.46	0.94 (0.87, 1.03)	0.16	**0.86 (0.75, 0.99)**	**0.04 **
LOT-R optimism subscale								
Model 1	0.96 (0.89, 1.04)	0.30	1.13 (0.95, 1.33)	0.17	0.91 (0.79, 1.04)	0.16	**0.77 (0.62, 0.95)**	**0.01 **
Model 2	0.98 (0.90, 1.07)	0.64	1.15 (0.97, 1.36)	0.12	0.92 (0.80, 1.07)	0.29	**0.75 (0.59, 0.95)**	**0.02 **
Model 3^*∗*^	0.99 (0.91, 1.08)	0.83	1.15 (0.97, 1.36)	0.11	0.94 (0.81, 1.08)	0.38	**0.78 (0.62, 0.98)**	**0.03 **
Model 4	1.00 (0.92, 1.09)	1.00	1.15 (0.97, 1.37)	0.11	0.94 (0.81, 1.08)	0.38	**0.77 (0.61, 0.97)**	**0.03**
Model 5	1.00 (0.92, 1.09)	1.00	1.16 (0.97, 1.38)	0.10	0.92 (0.80, 1.07)	0.30	**0.77 (0.61, 0.99)**	**0.04 **
LOT-R pessimism subscale								
Model 1	**1.16 (1.09, 1.25)**	**<0.001**	1.09 (0.95, 1.26)	0.23	**1.24 (1.10, 1.39)**	**<0.001**	**1.31 (1.08, 1.58)**	**0.007 **
Model 2	1.05 (0.97, 1.13)	0.23	1.03 (0.88, 1.19)	0.74	1.10 (0.96, 1.25)	0.16		
Model 3^*∗*^	1.05 (0.98, 1.13)	0.18	1.03 (0.89, 1.20)	0.71	1.11 (0.97, 1.26)	0.13	1.22 (0.98, 1.52)	0.07
Model 4	1.04 (0.97, 1.12)	0.27	1.02 (0.88, 1.18)	0.80	1.08 (0.95, 1.23)	0.23	1.18 (0.95, 1.47)	0.13
Model 5	1.04 (0.96, 1.12)	0.33	1.02 (0.88, 1.19)	0.79	1.10 (0.96, 1.25)	0.19	1.20 (0.95, 1.50)	0.13

Reference: (1) Model 1: it includes LOT-R, Model 2: it includes Model 1 + age, Model 3: it includes Model 2 + sex, Model 4: it includes Model 3 + lifestyle variables (average week alcohol use, smoking status, waist-to-hip ratio, and exercise status), Model 5: it includes Model 4 + medication variables (angina meds, cholesterol-lowering meds, and diabetic meds), and Model 6: it includes Model 5 + SF-12 variables (PCS, MCS). (2) All-cause mortality (*n* = 198), cancer mortality (*n* = 49), CVD mortality (*n* = 62), and CHD mortality (*n* = 22). (3) ^*∗*^LOT-R and sex interaction is not statistically significant. Bold indicates *p* value ≤ 0.05.
